# Cardioimmunology in Health and Diseases: Impairment of the Cardio-Spleno-Bone Marrow Axis Following Myocardial Infarction in Diabetes Mellitus

**DOI:** 10.3390/ijms252111833

**Published:** 2024-11-04

**Authors:** Amankeldi A. Salybekov, Kanat Tashov, Yin Sheng, Ainur Salybekova, Yoshiko Shinozaki, Takayuki Asahara, Shuzo Kobayashi

**Affiliations:** 1 Kidney Disease and Transplant Center, Shonan Kamakura General Hospital, Kamakura 247-8533, Japan; shuzo@shonankamakura.or.jp; 2 Shonan Research Institute of Innovative Medicine, Shonan Kamakura General Hospital, Kamakura 247-8533, Japan; 3 Department of Advanced Medicine Science, Tokai University School of Medicine, Isehara 259-1193, Japan; tashovkanat@gmail.com (K.T.); shengyin2017@gmail.com (Y.S.);; 4 Teaching and Research Support Core Center, Tokai University School of Medicine, Isehara 259-1193, Japan

**Keywords:** diabetes, myocardial ischemia, immune cell kinetics, cardio-spleno-bone marrow axis

## Abstract

A comprehensive understanding of the cardio-spleen-bone marrow immune cell axis is essential for elucidating the alterations occurring during the pathogenesis of diabetes mellitus (DM). This study investigates the dynamics of immune cell kinetics in DM after myocardial infarction (MI) over time. MI was induced in diabetic and healthy control groups using C57BL/N6 mice, with sacrifices occurring at days 1, 3, 7, and 28 post-MI to collect heart, peripheral blood (PB), spleen, and bone marrow (BM) samples. Cell suspensions from each organ were isolated and analyzed via flow cytometry. Additionally, the endothelial progenitor cell-colony-forming assay (EPC-CFA) was performed using mononuclear cells derived from BM, PB, and the spleen. The results indicated that, despite normal production in BM and the spleen, CD45+ cells were lower in the PB of DM mice at days 1 to 3. Further analysis revealed a reduction in total and pro-inflammatory neutrophils (N1s) in PB at days 1 to 3 and in the spleen at days 3 to 7 in DM mice, suggesting that DM-induced alterations in splenic neutrophils fail to meet the demand in PB and ischemic tissues. Infiltrating macrophages (total, M1, M2) were reduced at day 3 in the DM-ischemic heart, with total and M1 (days 1–3) and M2 (days 3–7) macrophages being significantly decreased in DM-PB compared to controls, indicating impaired macrophage recruitment and polarization in DM. Myeloid dendritic cells (mDCs) in the heart were higher from days 1 to 7, which corresponded with the enhanced recruitment of CD8+ cells from days 1 to 28 in the DM-infarcted myocardium. Total CD4+ cells decreased in DM-PB at days 1 to 3, suggesting a delayed adaptive immune response to MI. B cells were reduced in PB at days 1 to 3, in myocardium at day 3, and in the spleen at day 7, indicating compromised mobilization from BM. EPC-CFA results showed a marked decrease in definitive EPC colonies in the spleen and BM from days 1 to 28 in DM mice compared to controls in vitro, highlighting that DM severely impairs EPC colony-forming activity by limiting the differentiation of EPCs from primitive to definitive forms. Taking together, this study underscores significant disruptions in the cardio-spleen-bone marrow immune cell axis following MI in DM, revealing delayed innate and adaptive immune responses along with impaired EPC differentiation.

## 1. Introduction

Myocardial-infarcted (MI) tissue’s cellular content is indispensably required to fully comprehend the variations that occur during pathogenesis, which is crucial to developing proper medicine strategies. Myocardial infarction induces a complex inflammatory response that results in the recruitment and activation of the innate and adaptive immune systems [1]. This brisk inflammatory response upregulates a suite of cytoprotective and/or reparative responses that provide the heart with short-term adaptations to environmental stress [2]. However, this same inflammatory response often results in unintended collateral myocardial damage that can lead to adverse left ventricular (LV) remodeling and progressive dysfunction. This occurs in approximately 15% of patients following myocardial infarction (MI) advances such as a mechanical reperfusion strategy [3].

Researchers have shown that myeloid and lymphoid immune cell subsets infiltrate the ischemic myocardium, and their negative correlation with spleen immune cell number indicates the presence of the cardio-splenic axis [4,5]. Mounting reports indicating that pro-inflammatory neutrophils or macrophages, also known as N1s and M1s, accumulate in necrotic tissue to detect endogenous danger signals, clear debris, and regulate the inflammatory response via classical antigen-presenting cells like dendritic cells (DCs) [6]. By presenting MHC class I molecules to CD8+ cells (cytotoxic or killer T cells) or MHC class II molecules to CD4+ cells (helper T cells), dendritic cells (DCs) present antigens or activate an adaptive immune response [7]. During the reparative phase, alternatively activated neutrophils and macrophages, also known as N2s and M2s, secrete various reparative growth factors and cytokines to facilitate tissue healing [8].

The majority of the research studies have focused on the kinetics and dynamics of immune cells in infarcted myocardia or spleens, including neutrophils, macrophages, DCs, and T cells, in animal models [4,5,6]. However, there is lack of studies on the cardio-spleno-bone marrow axis in healthy and diabetic conditions in post-MI remodeling. The study aims to explore the changes in the innate and adaptive immune cell kinetics of diabetic animals’ ischemic myocardia, peripheral blood, spleens, and bone marrow (BM) at various time points after the onset of MI.

## 2. Results

### 2.1. Animal Characteristics

**Figure 1:**
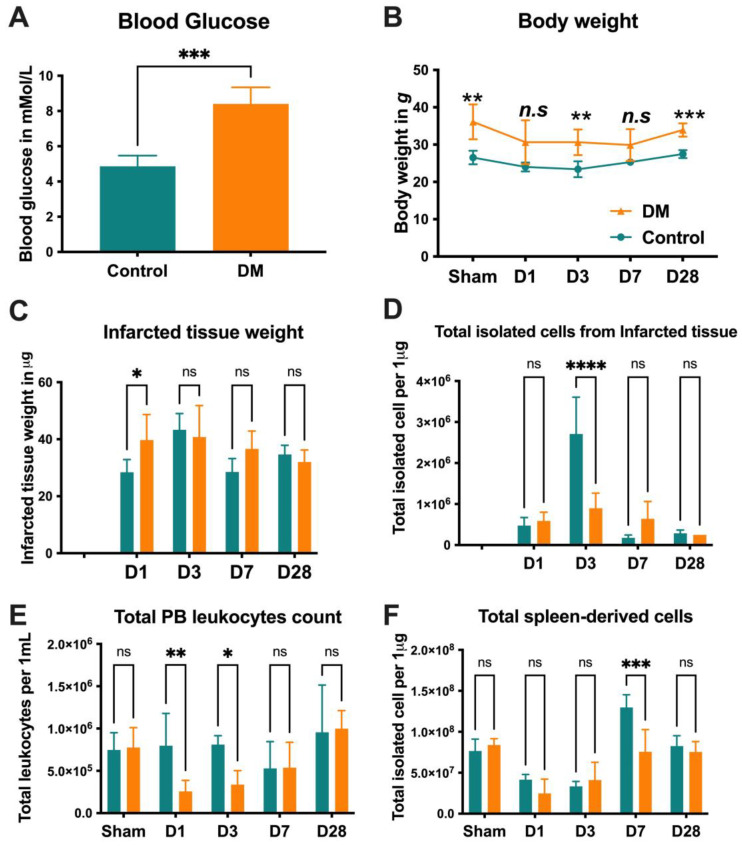
Group characteristics. The glucose levels and body weight of the diabetic (DM) group were significantly higher, confirming successful DM induction (**A**,**B**). The total number of isolated tissues and cells did not show significant differences, except for (**C**) the total amount of isolated cells from infarcted tissues on D3, (**D**) the total peripheral blood leukocyte count on D1 and D3, and (**E**) the total amount of spleen-derived cells on D7 (**C**–**F**). Data are represented as the mean ± SE. N = 10–12 mice per group. The experiments were repeated twice. In the graph, * *p* < 0.05, ** *p* < 0.01, *** *p* < 0.001, and **** *p* < 0.0001, ns/n.s means not significant, as determined by a Mann–Whitney test, Two-way ANOVA, followed by a Sidak or Benjamini’s multiple comparison test.

Based on our results shown in Figure 1A,B, the diabetic group exhibited significantly elevated glucose levels and BW compared with the control group, indicating successful diabetes induction in these animals. The total number of isolated cells from infarcted tissue on day 3 (D3), the total PB leukocyte count on day 1 (D1) and D3, and the total amount of spleen-derived cells on day 7 (D7) was significantly elevated in the control group in comparison to the DM group. (Figure 1C–F).

### 2.2. Neutrophils Kinetics After Onset MI

**Figure 2:**
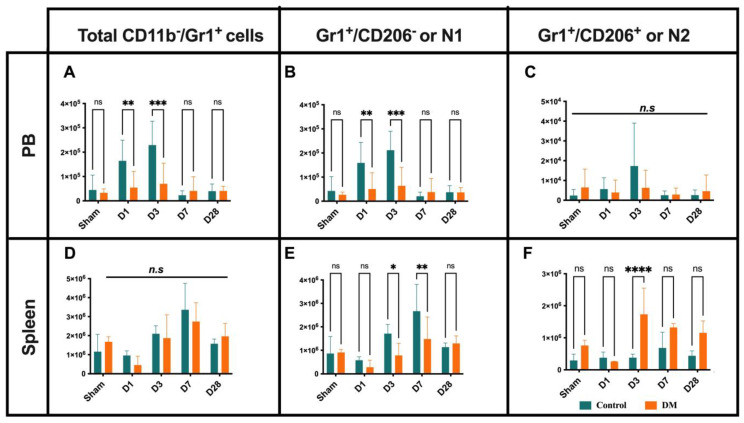
Neutrophil dynamics. The number of infiltrated total neutrophils and their N1 subset significantly increased at D1 and peaked at D3 in the PB of control mice (**A**,**B**) but insignificant in N2 subset (**C**). At D3 and D7, the total number of spleen-derived neutrophils did not changed (**D**) except N1 neutrophils gradually increased in the control group (**E**), whereas N2 subsets were enlarged in diabetes (**F**). Data are represented as the mean ± SE. N = six–eight mice per group. Experiments were repeated twice. In the graph, * *p* < 0.05, ** *p* < 0.01, *** *p* < 0.001, and **** *p* < 0.0001, ns/n.s means not significant, as determined by Two-way ANOVA, followed by a Sidak or Krieger and Yekutieli multiple comparison test.

Despite the mobilization of spleen CD45+ cells to peripheral blood (PB), the proportion of CD45+ cells in the diabetes group was significantly lower compared to the control group at D1 and D3. The production of CD45+ cells in the bone marrow (BM) increased equally in both groups. These findings suggest that CD45+ cell dynamics are altered in the spleen and PB during the acute phase of myocardial infarction in diabetic mice, even when BM production remains normal (Supplementary Figure S1). Next, we analyzed the dynamics of neutrophils’ immune response in relation to myocardium infarction (Figure 2). The number of infiltrated Ly-6G+ neutrophils, as well as their pro-inflammatory (N1) and anti-inflammatory (N2) phenotypes, did not show statistically significant differences between the control and diabetic groups. The total number of the neutrophil count in DM mice and their N1 subset was significantly lower at D1 and D3 in peripheral blood (PB) (Figure 2A–C). In the control group, N1 neutrophils in the spleen gradually increased on D3 and D7, whereas, in diabetic mice, there was an enlargement of the N2 subset (Figure 2D–F). These data may suggest that the diabetic condition itself induces spleen and BM neutrophil phenotype changes, and the latter may not compensate for PB and ischemic tissue demand.

### 2.3. Macrophages Dynamics

**Figure 3:**
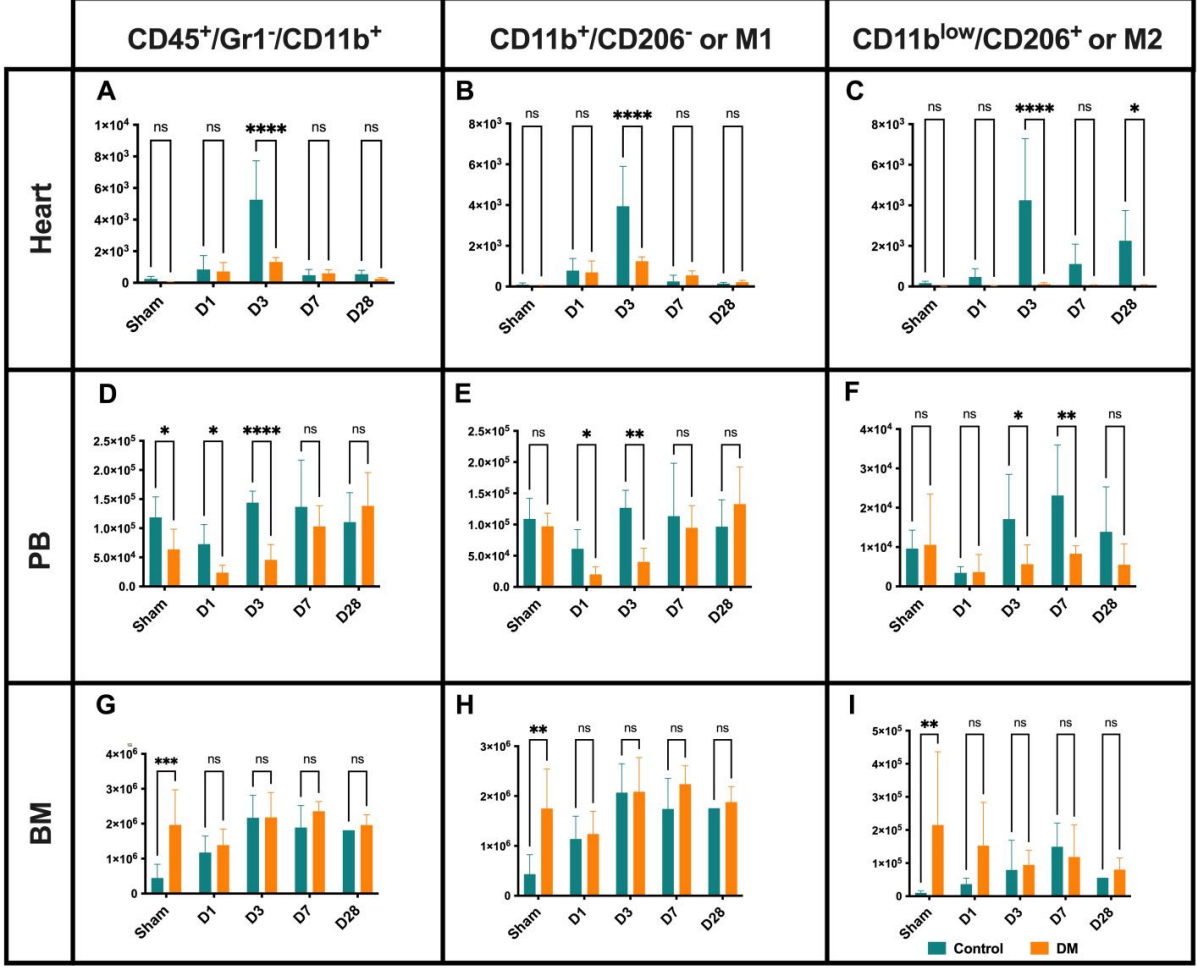
Macrophage dynamics. Infiltration of the M1 and M2 subsets into the ischemia tissue peaks at D3 in controls compared to DM (**A**–**C**). M2 macrophage recruitment demonstrate two waves on D3 and D28 (**C**). In control vs. DM PB, number of total and M1 macrophages increased from D1–D3, while the number of M2 cells increased from D3–D7 (**D**–**F**), indicating delayed and impaired macrophage infiltration, particularly M2 subset recruitment, in the ischemic myocardium of DM following MI. Sham group BM the total macrophage, M1, and M2 level are higher in DM groups (**G**–**I**). Data are represented as the mean ± SE. N = six–eight mice per group. Experiments were repeated twice. In the graph, * *p* < 0.05, ** *p* < 0.01, *** *p* < 0.001, and **** *p* < 0.0001, ns means not significant as determined by Two-way ANOVA, followed by a Sidak or Krieger and Yekutieli multiple comparison test.

Consistent with previous reports, macrophage infiltration, including M1 and M2 subsets, peaked on D3 post-MI in control groups compared to the diabetic groups (Figure 3A–C). Flow cytometry analysis revealed two distinct waves of M2 macrophage recruitment in the ischemic myocardium in control mice, occurring on D3 and day 28 (D28), whereas this recruitment was impaired in the diabetes group (Figure 3C). In the PB of the control versus diabetic mice, the total macrophage count and the M1 subset increased from D1 to D3, while M2 cell numbers rose from D3 to D7 (Figure 3D–F). The macrophage counts in the spleen and BM were similar between the two groups (Figure 3G–I). These results suggest that diabetic conditions lead to delayed and impaired macrophage infiltration, particularly in regard to the recruitment of the M2 subset, of the ischemic myocardium following MI.

### 2.4. Dendritic Cells Dynamics

**Figure 4:**
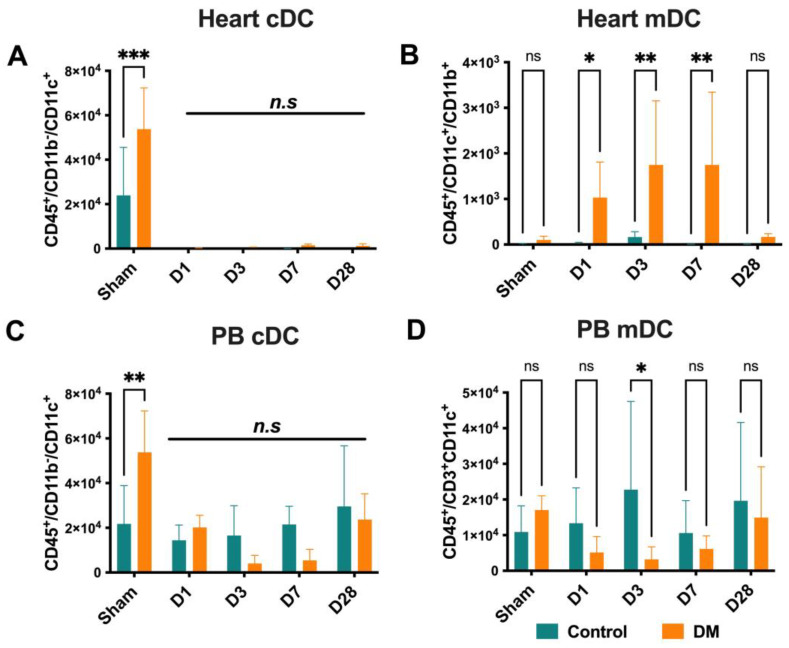
Dendritic cell dynamics. Although there were no significant differences observed in conventional dendritic cells (cDCs) in the heart and PB between DM and healthy mice (**A**,**C**), myeloid dendritic cells (mDCs) increased in the DM heart on D1-D7 (**B**). mDCs decreased on D3, indicating a possible mobilization of these cells from the PB to the heart during the acute phase of MI in DM (**D**). Data are represented as the mean ± SE. N = six–eight mice per group. Experiments were repeated twice. In the graph, * *p* < 0.05, ** *p* < 0.01, and *** *p* < 0.001, ns/n.s means not significant, as determined by Two-way ANOVA, followed by a Sidak or Krieger and Yekutieli multiple comparison test.

Based on our findings, although there were no significant differences observed in the conventional dendritic cells (cDCs) in the heart and PB between DM and healthy groups (Figure 4A,C), diabetic mice exhibited significant increases in myeloid dendritic cells (mDCs) in the heart on D1, D3, and D7. In PB, a dramatic decrease in mDCs was observed on D3, indicating a possible mobilization of these cells from the PB to the heart during the acute phase of MI in diabetic conditions (Figure 4B,D).

### 2.5. T Cells Distribution

**Figure 5:**
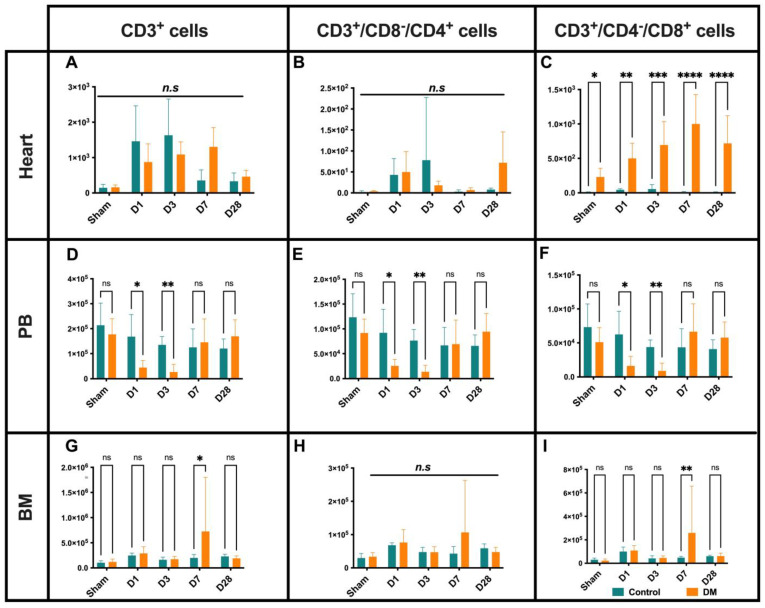
T cell dynamics: The total recruitment of T cells to the myocardium increased on D1 and peaked at D3 in control mice, while, in diabetes mice, the peak was observed on D7 (**A**). In the control group, the main infiltrated cells into the ischemic myocardium on D1-D3 were helper T cells, whereas cytotoxic T cell recruitment was significant in diabetics (**B**). Cytotoxic T cell numbers gradually increased in the heart tissues from D1–D28, even in sham-operated diabetic mice (**C**). The total T cell, T helper, and cytotoxic T cell levels in PB diminished in DM mice compared to the control group at D1–D3 (**D**–**F**). In D7, the subset of total and cytotoxic T cells in the BM of the DM group were higher than their healthy counterparts (**G**–**I**). Data are represented as the mean ± SE. N = 6–8 mice per group. Experiments were repeated twice. In the graph, * *p* < 0.05, ** *p* < 0.01, *** *p* < 0.001, and **** *p* < 0.0001, ns/n.s means not significant, as determined by Two-way ANOVA followed by a Sidak or Krieger and Yekutieli multiple comparison test.

The total CD3^+^ cell recruitment to the myocardium gradually increased from D1 and peaked at D3 in the control mice, whereas the total recruited CD3^+^ peak was observed at D7 in the diabetic mice, indicating a delayed adoptive immune response to the aseptic necrosis (Figure 5A). When we further dissected CD3^+^ cells into helper T cells (CD3^+^/CD8^−^/CD4^+^) and cytotoxic T cells (CD3^+^/CD4^−^/CD8^+^), we found that the main infiltrated cells into the ischemic myocardium on D1 to D3 in the control group were helper T cells, whereas CD8^+^ cell recruitment was significant in the diabetic group (Figure 5B,C). Notably, CD8^+^ cell numbers were gradually elevated in heart tissue from D1 to D28 (and even in the sham-operated diabetic mice) (Figure 5C). The PB total T cell, T helper, and cytotoxic T cell levels diminished in the diabetes mice vs. their control counterpart on D1 and D3 (Figure 5D–F). On D7, the total T cells and its cytotoxic subset were significantly more increased in the BM of the DM group than in their healthy counterpart group (Figure 5G–I). These findings indicate a delayed and altered recruitment pattern of T cell subsets in regard to the ischemic myocardium, as well as abnormal T cell subsets in BM and PB, providing additional evidence of immune dysregulation in diabetes.

### 2.6. B Cells Distribution

**Figure 6:**
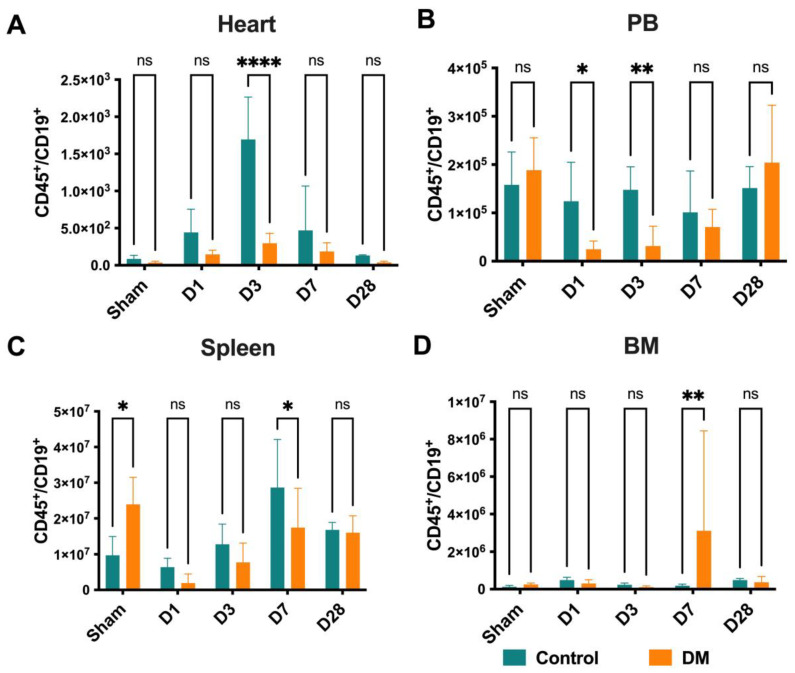
B cell dynamics: B cell infiltration into the ischemic heart peaked around D3 in the control group compared to the DM mice (**A**). The DM group exhibited significantly lower numbers of B cells in the PB compared to the control group on D1–D3 (**B**). Interestingly, in the sham-operated diabetes group, the B cell numbers in the spleen were higher than in the healthy group. After MI, the number of B cells in the control mice significantly increased compared to the DM mice on D7 (**C**). On D7, the number of BM-B cells in the DM group was higher than in the control group (**D**), suggesting an impaired B cell response in Dm mice. Data are represented as the mean ± SE. N = six–eight mice per group. Experiments were repeated twice. In the graph, * *p* < 0.05, ** *p* < 0.01, and **** *p* < 0.0001, ns means not significant, as determined by Two-way ANOVA, followed by a Sidak or Krieger and Yekutieli multiple comparison test.

CD19^+^ B cell infiltration into ischemic heart peaked around D3 in the control mice compared to their diabetic counterparts, then quickly decreased with only minimal CD19^+^ cells remaining at D7 or D28 post-MI (Figure 6A). The diabetes group’s PB CD19^+^ cell numbers were significantly lower in the acute phase of MI, on D1 and D3, when compared to the control group (Figure 6B). Interestingly, the spleen B cell numbers in the sham-operated diabetes group were higher than in the healthy control group. After MI, the number of B cells in the control mice significantly increased compared to the diabetes mice on D7 (Figure 6C). These data may indicate that the decreased number of B cells at D1 through D3 (due to mobilized into the PB) did not compensate at the same level as the healthy control group. Also, at D7, the number of BM B cells in the diabetes group was higher than the control group (Figure 6D). Our findings demonstrate an impaired B cell response in DM following MI.

### 2.7. Endothelial Progenitor Cells Colony Formation Assay (EPC-CFA)

**Figure 7:**
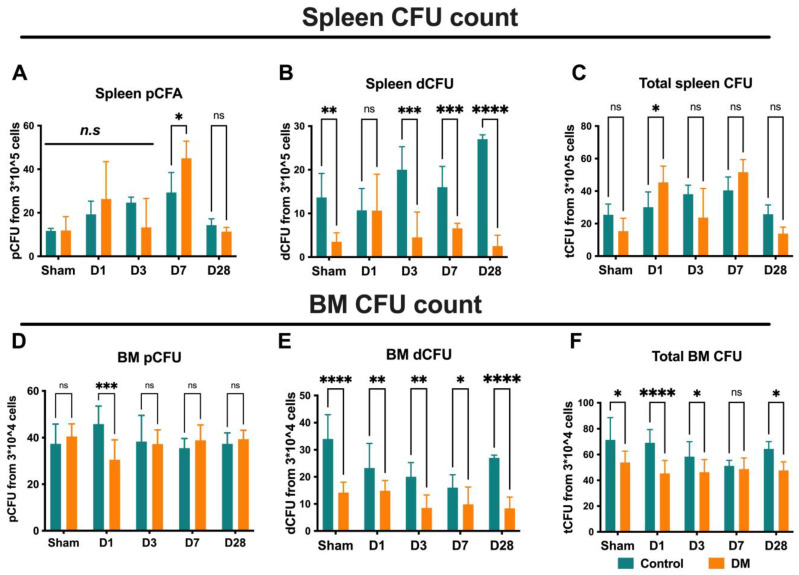
Endothelial progenitor cells colony formation assay (EPC-CFA) in the spleen and BM: In the spleen, the frequency of definitive EPC colonies (dEPC-CFUs) decreased in DM mice at D 3, D7, and D28 (**A**–**C**). In BM, dEPC-CFUs decreased in DM compared to control mice (**D**–**F**), suggesting that DM conditions significantly impair EPC colony-forming functionality by limiting EPCs’ differentiation from primitive to definitive. Data are represented as the mean ± SE. N = six–eight mice per group. Experiments were repeated twice. In the graph, * *p* < 0.05, ** *p* < 0.01, *** *p* < 0.001, and **** *p* < 0.0001, ns/n.s means not significant, as determined by Two-way ANOVA, followed by a Sidak or Krieger and Yekutieli multiple comparison test.

**Figure 8:**
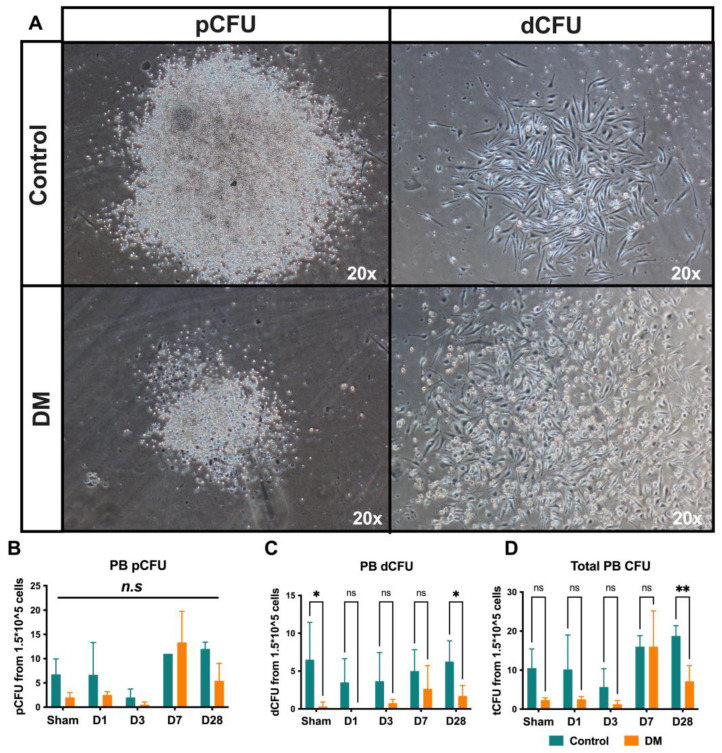
Endothelial progenitor cells colony formation assay (EPC-CFA) in PB: The primitive EPC colonies (pEPC-CFUs) have a lower density in the DM group, whereas, in the control group, the number of definitive EPC colonies (dEPC-CFU) was higher (**A**–**D**). Data are represented as the mean ± SE. N = six–eight mice per group. Experiments were repeated twice. In the graph, * *p* < 0.05 and ** *p* < 0.01, ns/n.s means not significant, as determined by Two-way ANOVA, followed by a Sidak or Krieger and Yekutieli multiple comparison test.

To verify the colony-forming capability, we performed EPC-CFA using BM, PB, and spleen-derived mononuclear cells. We did not assess EPC-CFU formation in cardiac cells because of contamination from cardiac endothelial cells, which can mimic the cobblestone-like morphology of EPCs. pEPC-CFUs are derived from relatively immature and highly proliferative EPCs, while dEPC-CFUs are relatively mature, differentiated, and able to promote the EPC-mediated cell functions required for vasculogenesis. As demonstrated in Figure 7A–F, the definitive or large-sized colonies (also known as mature EPC colonies) have a lower density in the diabetes group than the control group, indicating impaired colony-formation capabilities in DM animals. In the spleen, the frequency of dEPC-CFUs per 1 mL blood sharply decreased in the diabetes mice compared with the control mice on D3, D7, and D28 after the onset of MI (Figure 8A–C). Notably, the majority of diabetes group colonies were primitive, which is consistent with the previous report. In BM, we found a large number of dEPC colonies in controls versus diabetes (Figure 8A–D). These findings suggest that diabetic conditioning significantly impairs EPC colony-forming bioactivity by limiting EPCs’ differentiation from primitive to definitive.

## 3. Discussion

The kinetics and dynamics of cardio-spleno-bone marrow immune cells in diabetes following post-MI remodeling are not well understood. Our study demonstrated impairments in the kinetics of both adaptive and innate immune cells, as well as disruptions in their interactions after MI.

### 3.1. Neutrophils

One notable finding regarding neutrophil dynamics in our study is the decrease in total neutrophils and their N1 subset in the PB of diabetic mice by D1, peaking on D3, compared to their control counterparts. Bratton et al. [9] noted in a preclinical study that neutrophils were the first inflammatory cells to be recruited to the infarcted area, beginning to infiltrate within hours post-MI, peaking between D1 to D3, declining by day D5, and remaining at very low levels by D7 post-MI in animals without concomitant diseases [9]. Clinical findings were reported by Mangold et al. [10] that suggested that the neutrophils were highly activated in patients with ST-elevated acute coronary syndrome, serving as a predictor of ST-segment resolution and the size of the myocardial infarct. Another significant finding in neutrophil kinetics in diabetic animals is the gradual decrease in N1 neutrophils in the spleen on D3 and D7 compared to the control group. The N2 subset markedly increased on D7 under diabetic conditions. It is important to note that neutrophils, along with their respiratory burst, release various proteases and high levels of reactive oxygen species (ROS), which regulate LV remodeling by controlling cell infiltration and modulating biological molecules [10]. In this context, Ma et al. [11] demonstrated that neutrophils from the infarcted LV of mice exhibited N1 polarization on D1 (driven by damage-associated molecular patterns (DAMPs) and Toll-like receptor-4) and N2 polarization on D5 and D7 post-MI. Based on these observations, it can be inferred that neutrophils play a crucial role in managing the acute phase of LV remodeling and initiating an acute inflammatory response to clear dead cells and tissue debris, thereby contributing to post-MI repair. However, as we demonstrated, diabetic conditions may exacerbate the influx of N1 cells into the ischemic myocardium during the acute phase of MI, as well as in the spleen and bone marrow. Moreover, the latter may struggle to adequately compensate for the demands of ischemic tissue because most neutrophils have already shifted their phenotype from N1 to N2.

### 3.2. Macrophages

The kinetics of macrophages under ischemic conditions have been the focus of extensive research over the past two decades. The results demonstrate that infiltration of M1 and M2 macrophages into ischemic tissue peaks on day 3 following the onset of myocardial infarction in the control groups as compared to those with diabetes. Notably, the recruitment of M2 macrophages into the ischemic myocardium occurs in two distinct waves, observed on days 3 and 28, respectively. It has been believed that the macrophage phenotype transition from the pro-inflammatory M1 toward an anti-inflammatory M2 subtype is a key event in the regeneration process of an infarcted area [12]. Our findings are consistent with the previously reported biphasic myeloid cell response following MI, describing an early dominance of the M1 phenotype followed by an abundance of the M2 phenotype on D7 post-MI in healthy mice [13,14]. Interestingly, despite the elevated total and M2 macrophages in the sham diabetes group, and the subsequent equal production of macrophages (D1 to D28) from bone marrow, the PB level of total, M1, and M2 macrophages are significantly reduced in diabetic animals. The latter end up with a significant impairment of the total macrophage and it`s subset, such as with M1 recruitment into the ischemic myocardium and the phenotype change to M2. In relation to this matter, it has been confirmed that there is a notable decrease in the levels of M2 in diabetic patients compared to those without diabetes, and this is strongly associated with the presence of microangiopathy, which aligns with our own research findings [15]. Swirski et al. [16] mentioned that atherosclerosis, obesity, and diabetes disrupt the balance of Ly-6C^hi^ and Ly-6C^lo^ monocytes, which is needed for ideal tissue regeneration following MI. Furthermore, we showed the promoted M1 polarization of macrophages in PB of diabetic mice. Torres-Castro et al. [17] put a further validation badge to our findings, indicating that hyperglycemia has direct effects on the M1 polarization of circulating human macrophages. Also, Nagareddy et al. [18] showed that DM context promoted the overexpression of alarmin S100A8/A9 and accelerated the HSCs differentiation toward the myeloid lineage, including neutrophils, monocytes, and M1 macrophages. Furthermore, Monnerat et al. found that the diabetic environment compels resident macrophages to secrete IL-1β, which destabilizes the electrical activity of cardiomyocytes and increases the risk of ventricular arrhythmias [19]. It may be conceptualized that skewed macrophage polarization may contribute to increased inflammation and impaired tissue repair in the diabetic myocardium.

### 3.3. Dendritic Cells

Regarding DC kinetics, our results demonstrated a significant amount of cDC presence in sham-operated diabetes animals’ myocardia. Right after MI, all of the cDC cells were replaced by an abundant infiltration of mDCs into the ischemic heart in the acute and sub-acute phases. This phenomenon may explain why PB mDCs are recruited into the ischemia in the acute and sub-acute phases of MI by induction of the CD8 cells MCH-I in a dependent manner in diabetes animals. In this regard, Yilmaz et al. demonstrated that circulating mDCs are reduced in patients with AMI, while cDCs have not changed significantly, indicating mDC infiltration into the infarcted heart and their decrease in PB [20]. Also, it has been shown that permanent LAD ligation increased the DC numbers in the infarct border area and peaked from D7-14 post-MI in a rat experimental model [21]. Anzai et al. [22] claim that post-infarct DC activation is part of a protective repair response. Accordingly, Naito et al. clarified that mDCs have disastrous effects post-MI and worsen heart function [23]. These findings draw our attention to the idea that recruitment of mDCs to the infarcted heart exacerbates the cardiac regeneration process in diabetics. Further investigations are required to better understand the underlying mechanisms and causative link between increased mDCs and decreased infarct healing in diabetic conditions.

### 3.4. T Cells and Its Subsets

Regarding the T cell’s kinetics, our results indicate a delayed adoptive immune response to diabetic conditions. Another intriguing finding of the study was that, in the acute phase of ischemia, T helper cells were the most prevalent infiltrated cells in the healthy group, whereas cytotoxic T cells were the main infiltrated cells in diabetic group. Notably, cytotoxic T cells were the most recruited cells (in all time periods, and even in sham animals) into the ischemic myocardia of diabetic mice. Mechanistically, endogenous danger signals (aseptic necrotic tissue), also known as DAMPs, mostly activate mDCs, also engaging in the two functions of antigen presentation in the T cells and debris scavenging [24,25]. In our study, mDC cells’ elevated activation and increase can be explained by the strong stimulation induction of the MCH class I, which activated CD8 T cells. Liang et al. support our findings that the ischemic heart tissues of diabetics have more CD8+ T cells compared to normoglycemic counterparts [26], validating our results. Moreover, Santos-Zas et al. [27] revealed that, following AMI, infiltrated CD8+ T cells into the ischemic cardiac tissue secreted Granzyme B, causing cardiomyocyte apoptosis, adverse LV remodeling, and exacerbation of heart functions in an experimental pig model. They also discovered that depleting CD8+ T cells reduced inflammatory feedback and apoptosis in ischemic injured tissue, limiting myocardial injury and improving cardiac hemostasis, unearthing a deleterious role of CD8+ T lymphocytes following acute ischemia [28]. Of note, Ma et al. [28] clarified that cytotoxic T cells are required for infiltration and activation of M1 macrophages into the ischemic heart tissue, which is the hallmark of acute cardiac inflammatory response. Also, we found that all T cell subsets in PB decreased in the diabetic condition. On the other hand, the number of spleen T cells in the diabetes group dramatically was reduced on D1, and this reduction cannot compensate for the total number of T cells in the circulation. Regarding the effect of diabetic conditions on T cells, although one study reported that T cell exhaustion ameliorates cardiac remodeling in diabetic cardiomyopathy [29], there is no study directly revealing the role of T cells and their subpopulations in diabetic ischemic hearts, calling attention to the need for further studies.

### 3.5. B Cells

The role of adaptive immunity and the immune response mediated by B cells in myocardial recovery following AMI is not yet fully understood. However, recent studies, including our own findings, have shed some light on the infiltration and dynamics of B cells in the ischemic heart and their potential implications in the context of diabetes. Our results, in alignment with published articles, indicate that B cells are actively recruited to the site of injury to participate in the immune response in healthy animals. However, in diabetic mice, the extent of B cell infiltration appears to be diminished compared to the control group, indicating impaired recruitment or altered migration patterns in B cells in diabetic myocardia. Following the acute phase, the number of infiltrating B cells in the ischemic heart rapidly decreases during the subacute and chronic phases post-MI. This implies that the presence of B cells may be more transient and limited to the early stages of myocardial injury and inflammation. Further investigation is needed to determine the specific functions and contributions of B cells during this initial phase and whether their presence influences subsequent myocardial recovery and remodeling. Notably, our findings reveal that the diabetic group exhibits significantly lower numbers of CD19^+^ B cells in PB at D1 and D3 post-MI compared to the control group. This suggests that the diabetic condition may influence the mobilization of B cells from the spleen into the PB following myocardial injury. The reduced numbers of B cells in the PB of diabetic mice indicate an impaired compensatory response in comparison to the healthy control group. The decreased number of B cells observed in the PB of DM mice during the early stages after MI suggests a potential impairment in the immune response mediated by B cells in diabetic individuals. Therefore, the diminished B cell response in diabetic mice may have implications for the overall immune defense mechanisms and subsequent myocardial healing and recovery. Although the mechanisms of action of B cell–mediated tissue damage/regeneration after AMI have not been completely revealed, there are several mechanisms that have been suggested, including dysregulation of B cell subsets, deposition of IgM/IgG antibodies, and B cell-mediated macrophage recruitment [30]. It has been proposed that B reg subsets limited LV remodeling after AMI through decreasing CCR2-mediated macrophage recruitment and mobilization from the BM [31]. Mechanistic insights from mouse models demonstrated an arrest of BM B cell maturation and function 24 h post-MI. In this respect, Xu et al. [32] discovered a link between increased B cell counts and improved ejection fraction in MI patients treated with percutaneous intervention (PCI), while B lymphopenia was linked to heart failure. Infusion of BM B cells drastically enhanced heart function and decreased infarct size following acute MI. Concerning the effect of diabetic conditions on B cells, we are the first study that has examined B cell kinetics in diabetic AMI, and no study has directly revealed the role of B cells and their subpopulations in diabetic ischemic hearts.

### 3.6. Endothelial Progenitor Cells

It worth mentioning that pEPCs, also referred to as early or immature EPCs, constitute a less mature subset of EPCs, possessing the capacity to proliferate and migrate from the BM or other origins into the bloodstream. On the other hand, dEPCs, which have undergone further differentiation and are more specialized in their function, refer to a more mature and committed population of EPCs that possess the capacity to directly contribute to the formation and maintenance of blood vessels by differentiating into mature ECs, playing a crucial role in vascular development and repair [33]. In our study, dEPC-CFUs decreased in the diabetes group compared to the control counterpart. In the spleen, the frequency of dEPC-CFUs per 1 mL blood sharply decreased in the diabetes mice compared to the control mice on D3, D7, and D28 after the onset of MI. Notably, the majority of diabetes group colonies were primitive, which is consistent with the previous report [9]. In BM, we found a large number of dEPC colonies in controls versus those with diabetes. These findings suggest that diabetic conditioning significantly impairs EPC colony-forming bioactivity by limiting the EPCs’ differentiation from primitive to definitive. These results corroborate the ideas of Antonio et al. [34], who suggested the reverse relationship of EPCs with diabetes severity. They discovered a progressive decline in EPC count in AMI patients, from prediabetes to DM, also finding that the glycemic level is a determinant factor in regard to the quantity of circulating EPCs in the acute phase of AMI conditions. Indeed, they proposed that the spoiled feedback of EPC quantity in the acute MI was an early result in the DM background. Up until the current research, there have been few clinical studies that have examined the kinetics of EPC mobilization after AMI in DM disease [35,36,37]. In those studies, circulating EPCs levels were decreased in diabetics comparing to nondiabetic subjects on D1 after the onset of acute MI. Furthermore, compared to nondiabetic patients, diabetic patients had a delayed peak level of circulating EPCs (from D5 in nondiabetic patients to D7 in diabetic patients) because of impaired HIF/p-Akt/p-eNOS/MMP-9 signaling in the BM mobilization process of diabetics [38,39]. A meta-analysis of 28 studies, including 4155 patients, assigns the prognostic value of reduced circulating proangiogenic cells in diabetic conditions [39]. Consistent with these pre-existing studies, this study endorsed the concept that the quality and quantity of EPCs were extremely decreased in the acute phases post-AMI in diabetic conditions compared to healthy subjects.

## 4. Materials and Methods

### 4.1. Animal Sample Size and Randomization

All animal experiments were performed in accordance with the Tokai University School of Medicine Animal Care and Use Committee ethical approval # I-20056 and were based on the Guide for the Care and Use of Laboratory Animals (National Research Council, Tokyo, Japan). A total of 100 male C57BL/N6 mice at the age of 10 weeks, weighing 20–22 g, were obtained from Clea Japan (Yokohama, Japan). Animals were maintained under standard conditions (20 ± 2 °C, relative humidity 50–60%, and 12 h/12 h light/dark cycles) and were monitored daily by the Animal Support Center for Medical Research and Education at the Tokai University School of Medicine. The mice were acclimatized for a week. The Diabetes Complication Consortium protocol was used to induce type two diabetes in mice. Briefly, C57BL/N6 mice received a high-fat diet (HFD), which constituted 60% fat (Cat#., D12492, Research Diet Inc., New Brunswick, NJ, USA), plus a low dose of streptozotocin (Sigma, Burlington, MA, USA) of 50 mg/kg per mouse via intraperitoneal injection, as described earlier [39]. Control group C57BL/N6 mice received a standard rodent diet. The food intake of the mice was recorded every two days, and their body weight (BW) and blood sugar (BS) were measured. After four weeks of feeding with the respective diets, mice were divided into two groups (n = 50 each): control and DM, respectively. Figure 1 displays all of the animals’ characteristics overtime.

### 4.2. Myocardial Infarction Induction

For the MI model, the animals were anesthetized with 3–4% sevoflurane (Maruishi Pharmaceutical Co., Ltd., Tokyo, Japan), orally intubated, and respired using a rodent ventilator (Harvard Apparatus, Burlington, MA, USA), as reported earlier [40]. After a left-sided thoracotomy, myocardial ischemia was induced by permanent occlusion of the left anterior descending artery (LAD) with a 7-0 silk suture. The sham group underwent left-sided thoracotomy without LAD ligation. The thorax, muscles, and skin layers were separately closed with a 5-0 silk suture. After the completion of each experiment, the mice were sacrificed using overdose anesthesia at 1, 3, 7, and 28 days after MI or sham surgery, and peripheral blood (PB) was drawn via the apex of the heart, followed by systemic perfusion with heparinized PBS (Gibco, ThermoFisher Scientific, Whaltam, MA, USA) to exclude or minimize blood cell contamination; the organs were then excised for further cell isolation.

### 4.3. Preparation of a Single-Cell Suspension

Peripheral blood cells isolation: The peripheral blood mononuclear cells (PBMNCs), neutrophils, and granulocytes were isolated by the Ficoll gradient centrifugation procedure. Briefly, the blood was collected into a 5 mL BD flow tube containing 1 mL of each Histopaque 1.083 g/mL (Cat#10831-100ML, Sigma–Aldrich, Burlington, MA, USA) and 1.119 g/mL (Cat#11191-100mL, Sigma–Aldrich, Burlington, MA, USA). Residual red blood cells were removed using an RBC lysing buffer (BD., San Jose, CA, USA), and, after a serial centrifuging process, cells were isolated and counted.

Cardiac single-cell isolation: To effectively liberate cardiac muscle cell types, isolated hearts were finely minced using forceps into ~2 mm pieces and placed in 3 mL high-glucose DMEM with collagenase type II (500 U/mL) (Worthington Biochemical Corporation, Lakewood, NJ, USA) and Collagenase/Dispase (1 mg/mL) (Roche Diagnostics, Bazel, Switzerland). The tissue was incubated at 37 °C for 15 min with gentle rocking. Following incubation, a tissue digestion buffer with tissue clusters was triturated by pipetting and again incubated at 37 °C, then triturated twice more (45 min of total digestion time). The final trituration was conducted by pipetting at 37 °C with gentle agitation, as described by Pinto et al. in Protocol # 2 [41].

Spleen single-cell suspension preparation: The spleen was removed aseptically and transplanted into a 5 mL aseptic Eppendorf tube, followed by the addition of 2 mL of aseptic PBS into the EP tube. The spleen was gently ground with a sterile needle rod until it was white and transparent. The ground tissue solution was filtered through a 70 μm filter mesh cap (BD, San Jose, CA, USA), and the filtered spleen tissue cell solution was collected and transferred into a sterile test tube. The supernatant was discarded after centrifugation of the spleen tissue cell solution at 300× *g* for 5 min. Next, 3 mL of diluted red blood cell (RBC) lysis buffer (BD, San Jose, CA, USA) was added and mixed until the cells mixed well, and then the cells were incubated for 5 min at room temperature. Then, the samples were centrifuged at 300× *g* for 5 min, and 1 mL of sterile PBS/EDTA was added to stop lysis. Additionally, the cells were washed with PBS/EDTA at 200× *g* for 5 min twice prior to cell counting.

BM cells isolation: Using sterile conditions, the mouse hemi-femur and tibia were excised, and the muscles and residue tissues surrounding the femur and tibia were removed with sterile forceps and scissors before being flushed out into a new tube. Collected BM was centrifuged at 300× *g* for 5 min, and the supernatant was then discarded. Then, the suspended pellet was properly mixed with 2 mL of RBC lysis buffer (BD, CA, USA) and incubated for 5 min at room temperature. The PBS/EDTA was added to stop lysis, and the cells were centrifuged at 200× *g* for 5 min. Then, the pellet was resuspended with PBS/EDTA, passed through a 70 μm filter mesh cap (BD, CA, USA), and washed with PBS/EDTA at 200× *g* for 5 min two times prior to cell counting.

### 4.4. Flow Cytometry Analysis

The cell surface Fcγ receptors were blocked with mouse anti-Fcγ receptor (Biolegend Co., Ltd., San Diego, CA, USA) to reduce nonspecific binding of antibodies and left at 4 °C for 30 min, then washed twice with FACS buffer. Subsequently, cells were stained with the mixture of antibodies indicated in Supplementary Table S1 at 4 °C for 30 min before then being washed twice, as described previously. Flow cytometric analysis was performed on a BD FACS Verse and Fortessa (BD, CA, USA), and data were analyzed using FlowJo (Tree Star 10.2 version, BD, NJ, USA) and FCSExpress software version 6 (DevovaSoftwares Ltd., Pasadena, CA, USA).

### 4.5. EPC Colony-Forming Assay

Freshly isolated mouse PBMCs and mouse BM mononuclear cells (BMMNCs) were cultured in a semisolid methyl cellulose-based culture medium (MethoCult™ SF M3236, STEMCELL Technologies Inc., Vancouver, BC, Canada) containing 100 ng/mL stem cell factor (SCF), 50 ng/mL vascular endothelial growth factor (VEGF), 50 ng/mL basic fibroblast growth factor (bFGF), 50 ng/mL epidermal growth factor (EGF), 50 ng/mL insulin-like growth factor (IGF), 50 ng/mL interleukin-3 (IL-3) (these six proteins were purchased from Peprotech, Inc., Rocky Hill, NJ, USA), 2 IU/mL heparin (Ajinomoto Pharmaceutical Co., Ltd., Tokyo, Japan), 30% (*v*/*v*) fetal bovine serum (Nichirei Biosciences Inc., Tokyo, Japan), and penicillin/streptomycin (100 U/100 μg/mL; Gibco), as reported previously [42]. Cells were seeded at 1.5 × 105 cells/35 mm dish (BD Falcon, BD Bioscience, San Jose, CA, USA) and left in a humidified incubator with 5% CO_2_ at 37 °C until EPC colony formation. The number of adherent colonies on the dishes was counted between days 6–10 using a gridded scoring dish (STEMCELL Technologies Inc., Vancouver, BC, Canada) under a phase contrast light microscope (Eclipse TE3000; Nikon, Tokyo, Japan). Primitive EPC colony-forming units (pEPC-CFUs) and definitive EPC colony-forming units (dEPC-CFUs) were separately counted.

### 4.6. Statistical Analysis

All statistical analyses were performed using GraphPad Prism 9.3 (GraphPad Prism Software ver. 9.3, Inc., San Diego, CA, USA). All values are displayed as mean ± SE. A Mann–Whitney U test was used for two non-parametric groups. For multiple comparisons between groups at different time points, 2-way ANOVA was applied, followed by a Sidak, Benjamini, or Krieger and Yekutieli post hoc test. Data are represented as the mean ± SE. In the graph, statistical significance is presented as * *p* < 0.05, ** *p* < 0.01, *** *p* < 0.001, and **** *p* < 0.0001. *p* < 0.05 value was considered to indicate statistically significant differences.

## 5. Conclusions

In conclusion, our study investigated the dynamics and distribution of immune cells in the context of diabetes following MI. The findings provide valuable insights into the altered immune response and EPC functionality in diabetic individuals and shed light on the complex interplay between diabetes and the immune system during myocardial recovery. Firstly, the reduced recruitment and altered phenotypes of immune cells in the diabetic group suggest a dysregulated immune environment, contributing to the delayed adaptive immune response observed in diabetic individuals following AMI. The altered dynamics of immune cells in the spleen, PB, and BM of DM mice highlight the systemic impact of diabetes on immune responses ends up with low survival (Supplementary Figure S2). In our study, we found that the levels of mDC and CD8+ cells markedly increased in both cells’ subsets, with the latter proving to be a foe to myocardium regeneration rather than a friend. Furthermore, our study revealed the significant impact of diabetes on EPC-mediated vasculogenesis. The diabetic condition impaired the colony-forming bioactivity of EPCs and limited their differentiation from primitive to definitive states. This impaired EPC function and differentiation capacity likely contributed to the impaired vascular repair and regeneration observed in diabetic individuals following acute MI. Future research should focus on unraveling the underlying molecular mechanisms and identifying potential therapeutic targets to mitigate the adverse effects of diabetes on immune responses and promote myocardial healing and recovery.

Overall, our study contributes to the growing body of knowledge regarding the complex interactions between diabetes, the immune system, and EPCs in the context of myocardial ischemia. The findings underscore the need for further investigations to elucidate the underlying mechanisms and develop interventions aimed at enhancing vascular repair, restoring EPC functionality, and improving outcomes in diabetic patients with myocardial ischemia.

## Data Availability

All data generated or analyzed during this study are included in this published article.

## Supplementary Materials

The following supporting information can be downloaded at: https://www.mdpi.com/article/10.3390/ijms252111833/s1.
