# Supplementary material for: Cardioimmunology in Health and Diseases: Impairment of the Cardio-Spleno-Bone Marrow Axis Following Myocardial Infarction in Diabetes Mellitus
Source: Int J Mol Sci. 2024 Nov 4;25(21):11833. doi: 10.3390/ijms252111833 (PMC11546687; doi:10.3390/ijms252111833)
Supplement: Supplementary file 1 [file ijms-25-11833-s001.zip › ijms-3239817-supplementary.pdf]

Supplementary file 1. Cardioimmunology in health and diseases: impairment of cardio-spleno-bone marrow axis following myocardial infarction in diabetes mellitus

Supplementary Fig S1.

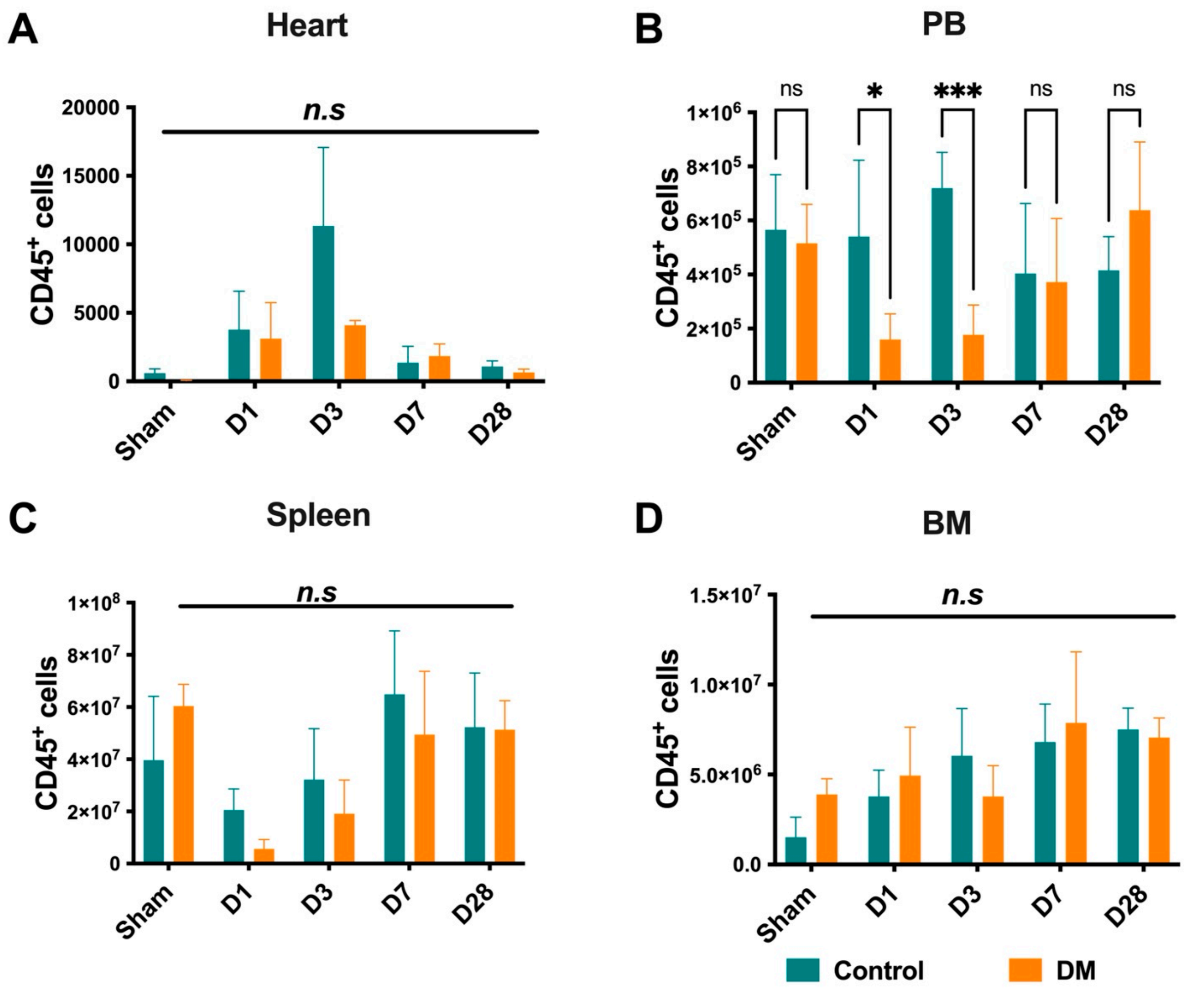

Supplementary Fig S1. A) Total infiltrated $CD45^+$ cells into the infarcted myocardial tissue. **B)** Peripheral blood total $CD45^+$ dropped at days one and three in DM group. **C)** and **D)** The total amount of $CD45^+$ cells change were insignificant in spleen and bone marrow. Data are represented

as the mean ± SE. N = 6-8 mice per group. Experiments were repeated twice. In the graph, *P < 0.05 and ***P < 0.001.

Supplementary Fig S2.

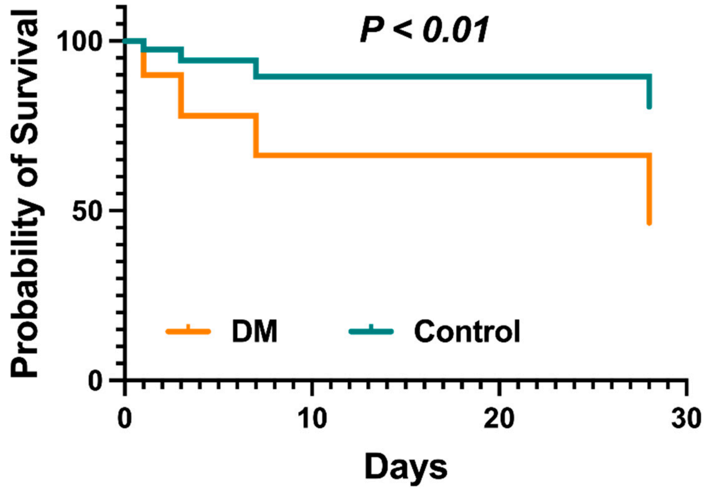

Supplementary Fig S2. Survival analysis. The Kaplan Meier statistical survival method was used.

Supplementary Table 1.

Table S1. Flow cytometry fluorophores. Prior to initiating the experiments, we thoroughly tested the compensation matrix for all antibodies and replaced any non-specific staining clones with alternative clones (Salybekov et al., 2019, PLOS ONE). The antibodies for T cells and B cells were prepared as mixtures, while a separate mixture was utilized for granulocytes and myeloid cells, with a defined gating strategy. Additionally, the staining mixtures for stem/progenitor cells and endothelial cells were kept distinct. Each mixture of antibodies included its respective IgG controls.

Antibodies	Clone	Manufacturer
anti-CD34	RAM34	Biolegend, San Diego, CA., USA
anti-CD45	30-F11	Biolegend, San Diego, CA., USA
anti-CD11b	M1/70	Biolegend, San Diego, CA., USA
anti-CD206	C068C2	Biolegend, San Diego, CA., USA

anti-CD11c	6D5	Biolegend, San Diego, CA., USA
anti-F4/80	BM8	Biolegend, San Diego, CA., USA
anti-LyG	RB6-8C5	Biolegend, San Diego, CA., USA
anti-CD31	390	Biolegend, San Diego, CA., USA
anti-NK-1.1	PK136	Biolegend, San Diego, CA., USA
anti-CD117	ACK2	Biolegend, San Diego, CA., USA
anti-CD184	L276F12	Biolegend, San Diego, CA., USA
anti-Ly6A/E (Sca-1)	D7	Biolegend, San Diego, CA., USA
anti-CD3e	145-2C11	Biolegend, San Diego, CA., USA
anti-CD4	GK.1.5	Biolegend, San Diego, CA., USA
anti-CD8a	53-6.7	Biolegend, San Diego, CA., USA
anti-CD25	PC61	Biolegend, San Diego, CA., USA
anti-CD127	A7R34	Biolegend, San Diego, CA., USA
